# Impact of a health services innovation university program in a major public hospital and health service: a mixed methods evaluation

**DOI:** 10.1186/s43058-022-00293-3

**Published:** 2022-04-25

**Authors:** Elizabeth Martin, Olivia Fisher, Gregory Merlo, Pauline Zardo, Sally E. Barrimore, Jeffrey Rowland, Janet M. Davies

**Affiliations:** 1 Australian Centre for Health Services Innovation (AusHSI), Centre for Healthcare Transformation, School of Public Health and Social Work, Queensland University of Technology (QUT), Brisbane, Queensland Australia; 2 Primary Care Clinical Unit, School of Clinical Medicine, University of Queensland (UQ), St Lucia, Queensland Australia; 3 Nutrition and Dietetics Department, The Prince Charles Hospital, Metro North Health, Chermside, Queensland Australia; 4 Internal Medicine, The Prince Charles Hospital, Metro North Health, Chermside, Queensland Australia; 5 Metro North Health, and Faculty of Health, Queensland University of Technology, Brisbane, Queensland Australia

**Keywords:** Evaluation, Health service research, Innovation, Implementation science, Evidence-based practice, Capacity-building, Knowledge translation

## Abstract

**Background:**

While health services and their clinicians might seek to be innovative, finite budgets, increased demands on health services, and ineffective implementation strategies create challenges to sustaining innovation. These challenges can be addressed by building staff capacity to design cost-effective, evidence-based innovations, and selecting appropriate implementation strategies. A bespoke university award qualification and associated program of activities was developed to build the capacity of staff at Australia’s largest health service to implement and evaluate evidence-based practice (EBP): a Graduate Certificate in Health Science majoring in Health Services Innovation. The aim of this study was to establish the health service’s pre-program capacity to implement EBP and to identify preliminary changes in capacity that have occurred as a result of the Health Services Innovation program.

**Methods:**

A mixed methods design underpinned by the Consolidated Framework for Implementation Research informed the research design, data collection, and analysis. Data about EBP implementation capacity aligned to the framework constructs were sought through qualitative interviews of university and health service executives, focus groups with students, and a quantitative survey of managers and students. The outcomes measured were knowledge of, attitudes towards, and use of EBP within the health service, as well as changes to practice which students identified had resulted from their participation in the program.

**Results:**

The Health Services Innovation program has contributed to short-term changes in health service capacity to implement EBP. Participating students have not only increased their individual skills and knowledge, but also changed their EPB culture and practice which has ignited and sustained health service innovations and improvements in the first 18 months of the program. Capacity changes observed across wider sections of the organization include an increase in connections and networks, use of a shared language, and use of robust implementation science methods such as stakeholder analyses.

**Conclusion:**

This is a unique study that assessed data from all stakeholders: university and health service executives, students, and their managers. By assembling multiple perspectives, we identified that developing the social capital of the organization through delivering a full suite of capacity-building initiatives was critical to the preliminary success of the program.

**Supplementary Information:**

The online version contains supplementary material available at 10.1186/s43058-022-00293-3.

Contributions to the literature

- Prior to program implementation, individual capacity to drive innovation and improvement across the health service was limited in scope and there was little cohesive organizational social capital.
- Critical elements influencing the preliminary success of the Graduate Certificate in Health Services Innovation program aligned with all five Consolidated Framework for Implementation Research constructs.
- Viewed together, these critical elements produced an emerging social capital within the health service that is supportive of innovation and evidence-based practice.
- Sustaining and expanding on the increased individual and organizational capacity to obtain a critical mass was acknowledged to be a long-term process.

## Background

Enthusiastic health service innovators are often “keen amateurs” who want to improve health systems [1]. They may exist within “pockets” of implementation science expertise with minimal capacity for supporting other’s enthusiasm. Both groups represent latent potential for health innovation and leadership [1, 2]. Innovators in health are critical because there is an increasing demand for access to healthcare internationally, and the costs of healthcare are rising faster than gross domestic product in many countries [3, 4]. Health services need to find sustainable and efficient ways of delivering care, and provide equitable access for the community through innovation [5, 7]. Despite the best intentions of keen amateurs who have not been supported by implementation scientists, many innovative projects fail or are not sustained [7, 8]. Supporting health service staff to pursue innovation and improvement is essential to solving these challenges.

Keen amateurs can be transformed into “hybrid clinicians,” or hybrid health service professionals, merging the worlds of healthcare, leadership, and research [9, 10]. The aim is to increase the use of evidence-based practices (EBP) such as the use of implementation science theories, models and frameworks [7], robust cost-effectiveness evaluation methods [11], and application of health systems research in health innovation and improvement as well as the use of clinical evidence [12]. Applying EBP methods is therefore not limited to research evidence informing *clinical care*, but also requires research to inform the implementation and evaluation of *improvements and innovations* that are beyond usual clinical care. To do this, healthcare organizations need to facilitate staff hybridity and leadership capabilities by taking both an individual support and organizational social capital approach [13] to develop a critical mass of staff using robust EBP methods informing all types of health service innovations. A social capital approach is when social relationships are valued and act as a resource that supports and enables production of benefits within and beyond a social group or network, such as clinicians in a health service. What is required are training programs that build individual skills and knowledge, complimented by a focus on shared leadership within the organization to facilitate the building of relationships, networking, trust, and commitment to the organization, as well as appreciating the social and political context [13–15].

Training impact has been primarily measured in terms of student projects commenced or completed, successful research grants, publications, and conference presentations [16, 17]. In one small study, organizational culture changes were described as increased preparation prior to innovation implementation such as training, communication, and a heightened awareness of how important implementation planning is [18, 19]. Few studies have used a control group to robustly evaluate training program impact [16]. Perspectives of participants’ managers or other health service managers not exposed to the various programs have not been examined at all. It remains unclear whether a capacity building training program can develop a critical mass of clinical and non-clinical staff using robust approaches to implementing EBP and driving innovation.

A collaborative team from a university and Australia’s largest publicly funded hospital and health service developed a bespoke post-graduate university program: the Graduate Certificate in Health Science (Health Services Innovation), hereafter known as “the program” [20]. The program was designed to increase capacity to implement both evidence-based methods and practice in healthcare by building individual skills and a critical mass of innovation social capital within the health service. A detailed description of the program and associated activities is in Additional file 1.

The aim of this research was to establish the health service’s pre-program capacity to implement EBP and to identify preliminary changes in capacity that have occurred as a result of the program. The research questions were as follows:

1. What was the health service capacity to implement EBP prior to commencement of the program?
2. Did the facilitation of the program improve implementation of EBP within the health service?
3. What are the contextual enablers and barriers to the program having an impact on the implementation of EBP?

Health services and universities can use our results from the first 2 years of the program to inform other investments and partnerships such as ours.

## Methods

### Design

The mixed methods evaluation design used the Consolidated Framework for Implementation Research (CFIR) [7, 21–25]. CFIR is a widely used framework designed to guide and inform implementation planning and evaluation [24] (Table 1). CFIR can be used pre-, during, or post-implementation. This study was conducted during the implementation of the program, and therefore, we have used CFIR to evaluate the drivers of implementation capacity, rather than determinants of post-implementation outcomes such as health service efficiency and patient outcomes [7]. CFIR constructs were used to guide the data collection tools such as development of the interview and focus group guides and also to guide analysis. In Table 1, we have provided examples of CFIR constructs that we sought to explore through the evaluation. As such, a constructivist research paradigm was used. The scope and setting for our research methods are described in Additional file 2.

**Table 1:** Consolidated Framework for Implementation Research (CFIR) constructs and methods

CFIR construct	Construct example	Data source	Analysis methods	Research question
Characteristics of the intervention	Adaptability, intervention source	• Interviews with university and HHS executives • Focus groups with cohort one students	Thematic analysis grouped by participant type; post hoc mapping of themes to CFIR construct (See Additional files 5, 6, and 7)	3. What are the contextual enablers and barriers to the program having an impact on the implementation of EBP?
Outer setting	National and state government health policies, incentives, funding	• Interviews with university and health service executives	As above	
Inner setting	Culture, relative priority, learning climate	• Interviews with university and health service executives • Focus groups with cohort one students • Surveys with students, managers of students, and control managers	As above	1. What was the health service capacity to implement EBP prior to commencement of the program? 2. Did the facilitation of the program improve implementation of EBP within the health service? 3. What are the contextual enablers and barriers to the program having an impact on the implementation of EBP?
Characteristics of individuals	Knowledge and beliefs, personal attributes	• Focus groups with cohort one students • IEBP survey with students, managers of students, and control managers	As above Descriptive analysis of quantitative indicators of barriers and enablers; thematic analysis of open field comments in survey (See Additional files 3, 4 and 7)	
Process	Planning, engagement, reflection, evaluation	• Interviews with university and HHS executives • Focus groups with cohort one students	As above	2. Did the facilitation of the program improve implementation of EBP within the health service? 3. What are the contextual enablers and barriers to the program having an impact on the implementation of EBP?

*HHS* hospital and health service, *EBP* evidence based-practice, *IEBP* implementing evidence-based practice

### Participants and recruitment

Five groups of participants were invited to participate in the mixed methods data collection approaches: members of both the university and health service executive leadership (*N*=9), program students, who were mostly senior clinicians employed within the health service (*N*=60), students’ managers (*N*=61), and a control group of managers (*N*=60) who did not have exposure to the program. All participants were purposefully sampled and recruited due to their direct or indirect involvement in the program. The control group of managers was matched to the roles (medical, nursing, allied health, or administration) of the students’ managers. Recruitment and data collection occurred between June and October 2019, 18 months after program delivery had commenced. In this short report, the methods of the executive and students’ managers interviews, and student focus groups are presented, while the validated Implementing EBP (IEBP) survey methods are available in Additional files 3 and 4 and not described, nor results reported below.

### Data collection

Structured interviews with members of the health service and university executive leadership were conducted to identify their perceptions of changed capacity across the organization. Interview questions are in Additional file 5.

Semi-structured focus groups were conducted with students from the first cohort to identify preliminary changes in health service capacity during their 18 months of participation. The focus group guide is available in Additional file 6, and the data collection procedures are outlined in Additional file 7.

### Data analysis and measurement

Interview and focus group transcriptions were analyzed thematically. For each dataset, two researchers conducted the thematic analysis through two examinations of the data. All four researchers then discussed and agreed to the final themes and sub-themes presented. These themes and sub-themes were mapped against CFIR constructs and sub-constructs by identifying key words and phrases in each theme group that gave further meaning to each CFIR construct. Doing this enabled us to identify which aspects of the program influenced its implementation and thus contributed to changes in EBP capacity across the health service [24]. The sources of data and analysis methods that would provide meaning for each identified construct, and the research questions that each method aimed to answer are summarized in Table 1.

Reporting standards adhered to relevant checklists (TIDIeR, SRQR guideline, and the STROBE checklist). See Additional files 8, 9, and 10.

## Results

### Participants

Three university and four health service executive staff who were involved in establishing the program were interviewed; a further two declined to participate. Eleven students participated in the focus groups: a response rate of 40.7% amongst students. Nine participated across three focus groups (*n* = 4, 3, and 2 participants in each). Two students were interviewed independently using the focus group questions as they were not able to attend any focus group times. None of the students’ managers who were approached for interviews agreed to participate. In this short report, we present a summary of qualitative results, while detailed results from both the qualitative and quantitative data can be found in Additional file 11.

### Themes

Four overarching themes emerged from the qualitative data: realization of knowledge gaps; increased individual and network capacity; promising, but early days; and organizational support in theory, barriers in practice. In Table 2, we have mapped the overarching themes and sub-themes from the executive interviews and student focus groups to CFIR constructs and identified which research questions the themes assist in answering.

**Table 2:** Summary of executive interview and student focus group themes describing program implementation and research question address, mapped to Consolidated Framework for Implementation Research (CFIR) constructs and subconstructs

CFIR constructs	Theme	Sub-theme summary (research question number)
**Characteristics of intervention**		
Intervention source	Organizational support in theory, barriers in practice	Breaking boundaries between healthcare and academic settings (3)^a^
Adaptability	Organizational support in theory, barriers in practice	Responsiveness to needs of students (3)^a^
**Outer setting**		
Cosmopolitan	Organizational support in theory, barriers in practice	Interconnectedness with university (3)^a^
**Inner setting**		
Networks and communications	Increased individual and network capacity	Relationships with cohort network (2)^b^
	Organizational support in theory, barriers in practice	Facilitation of greater interconnectedness (1, 2)^a^
	Promising, but early days	Developing a shared language (1, 2)^b^
Culture	Realization of knowledge gaps	Existing culture of improvement and EBM (1, 2)^a^
		Difficulty of culture change (3)^a^
	Promising, but early days	Culture change has already been experienced (2)^b^
Implementation climate	Realization of knowledge gaps	Executives recognize need for change (3)^a^
	Organizational support in theory, barriers in practice	Inelasticity of workforce (3)^a^
	Promising, but early days	Influence on other projects (2)^b^
Readiness for implementation	Organizational support in theory, barriers in practice	Leadership engagement–objectives of executives (3)^ab^
		Support and quality of leadership (3)^ab^ Available resources (3)^b^
**Characteristics of individuals**		
Knowledge and beliefs about the intervention	Increased individual and organization/network capacity	Enthusiasm of course participants (2, 3)^a^
		Realistic understanding of challenges regarding sustainability (2)^b^
Self-efficacy	Realization of knowledge gaps	Limited academic evaluation/implementation knowledge (1)^a^ Using new skills (2)^b^ Confidence and credibility (2)^b^
Individual stage of change	Realization of knowledge gaps	Self-reflection on previous experiences (1)^b^
Individual identification with organization	Realization of knowledge gaps	Frustration with slow pace of change (1, 2)^b^
Other personal attributes	Realization of knowledge gaps	Identified gaps in stakeholder engagement (1, 2)^b^
**Process**		
External change agents	Organizational support in theory, barriers in practice	Responsiveness of university in delivering bespoke program (3)^a^
Innovation participants	Increased individual and network capacity	Enthusiasm and successful engagement of students (3)^a^

Subthemes sourced from ^a^executive interviews and ^b^student focus groups

### Realization of knowledge gaps

Participants across all groups reported that prior to the program, health service staff were “doing” EBP, but exposure to the program facilitated realization of knowledge gaps, particularly around evidence for implementation and evaluation methods.

So, prior to this I thought I was ticking the box. I now know I wasn’t ticking the box ... (student focus group 2)

### Increased individual and network capacity

Students and executives both thought that the individual capacity of students, as well as the organization capacity, had increased as a direct result of their participation in the program. Executives observed an increase in knowledge and culture change within the student group in terms of enthusiasm and improved implementation planning:

[Referring to a feedback workshop with students] … it was one of the most extraordinarily positive upbeat, excited groups of people that I have come across. They were fighting with each other to tell us how excited they were with what they’d done and what they’d achieved… (executive interview 1)

### Promising, but early days

All participants consistently expressed that the preliminary impact of the program was promising but acknowledged that it was a long way from achieving the desired large scale culture change:

*…* trying to turn this organization into doing something efficiently is like turning an aircraft carrier. It is not going to happen overnight. (executive interview 3)

### Organizational support in theory, barriers in practice

Within the health service, participants across all groups, regardless of their exposure to the program felt that executive leadership support for EBP was a key enabler of the use of EBP within the service. However, substantial barriers to the use of robust EBP processes were reported in practice:

So, I think our innate sort of conservative nature, by and large as a workforce, means we don't adopt change easily. (executive interview 3)

Dominant CFIR subconstructs that enabled improved capacity sat within the inner setting and characteristics of individuals (Table 2). Networks and communication were facilitated by the exclusive cohort design of the program. Each group of students commencing annually attended the same program of study over 2 years and disseminated a shared language and shared innovative approaches through greater interconnectedness. The existing culture of improvement and EBP within sections of the health service facilitated further culture change, and the implementation climate was prime for a shift, although participants recognized how difficult this would be across the whole organization. Students and executives agreed that leaders in the health service were ready for implementation of the program, were well engaged, and provided sufficient direct program resources. Individuals’ beliefs about the intervention were enthusiastic as well as realistic in terms of what could be achieved in the first few years. Students developed self-efficacy in evaluation and implementation knowledge and skills. Some students had increased confidence, and many felt their credibility within the organization had improved following the program. It was critical for the university to build students’ confidence by creating time for students to self-reflect on their increased individual capacity through assessment feedback and individual support activities. However, their developing confidence came with concerns about how much the health service executives expected of them, as students were far more conscious now of their knowledge gaps. Realization of knowledge gaps amongst students and across parts of the health service was a key enabler for the preliminary improvements in organizational capacity to implement EBP.

## Discussion

In this paper, we identified that a unique Health Services Innovation program, which is the first of its kind in Australia, can contribute in the short-term to improvements in individual and health service capacity to implement EBP (See Additional file 11). Prior to program commencement, students reported that they were not conscious of knowledge gaps in implementation and evaluation and therefore thought that they were using EBP approaches effectively. The health service’s pre-program capacity was limited to small pockets of skilled implementation scientists and evaluators, with limited cost-effectiveness analysis knowledge. This individual capacity within the health service was previously not able to drive innovation and improvement across the health service because there was no cohesive organizational social capital. Facilitation of the program appears to have improved short-term capacity to implement EBP as participating students have not only increased their individual skills and knowledge, but also changed their EBP culture and practice which has ignited health service innovations and improvements in the first 18 months of the program. Students’ projects have included telehealth initiatives, supporting prescribing in primary care and new models of maternity care. Facilitators of these changes include an increase in connections and networks, use of a shared language, and use of robust implementation science methods such as stakeholder analyses. Sustaining and expanding on the increased individual and network capacity to obtain a critical mass were acknowledged to be a long-term process.

All participants agreed that executive support of the program was a key enabler of EBP at the health service. It could be argued that our theme *organizational support in theory but barriers in practice* is at the heart of dissemination and implementation practice and research. Leadership support alone is insufficient to ensure effective EBP implementation, and additional barriers must be overcome to ensure implementation success [25]. Our theme of *increased individual and network capacity* also aligns with previous research on capacity building training [17]. Through executive leadership, and practical support from coordinators embedded within the health service and university, the program has been able to take both an individual and social capital approach to building health professional leadership, embracing hybridity. It is this kind of shift that develops the social capital of the organization [26].

The strengths of this study are that we have built upon the results of noteworthy research [27] which emphasized how little evidence there is documenting the impact or effectiveness of educational interventions. We have proposed methods that may assist other researchers to have greater analytical rigor in educational research. A mixed methods approach should be an essential component of further designs, as should a control group, although we acknowledge that engagement of this control group may be challenging. In our study, we also contribute to identifying how educational interventions such as our program work, and under which circumstances: transformative professional education that harnesses flows of educational content and innovation. In this preliminary evaluation of the program, we have demonstrated that this can be done through a dynamic and agile education system, mentoring, workplace-based projects, health service incentives, and the completion of an accredited university award course [27, 28].

Key limitations of this study are that we did not collect baseline data until after the program had commenced, and none of the students’ managers who were approached for interviews agreed to participate and the IEBP survey had a low response rate (See Additional file 11). Middle managers are often difficult to engage in innovation [29, 30]. Middle managers may have been disengaged in our research about innovation in their health service because of the absence of a “road” or organizational structure that connects them to both practice and executive support [30, 31]. Middle managers are largely overlooked in healthcare innovation, and we suspect we unfortunately also did this with our research engagement strategies [30]. In the IEBP survey, the low response rate may also be due to the difficulties engaging people who are not directly involved in the program. Nevertheless, there was a high level of engagement from both students and the executive team in the focus groups and interviews providing a rich source of data for analysis.

## Conclusions

Building implementation skills and social capital within a health service through educational programs is novel, but critical to sustaining health improvements and innovations [26, 32]. We have demonstrated that what is required for short-term improvements in capacity are leadership of an innovative workplace culture, skilled-up health staff with the mandate to be innovative, and the facilitation of a well-resourced organizational structure which interfaces between different parts of the health service, including middle management [29, 33, 34]. Our approach to education has created an environment in which health service innovators can thrive [35]. It is this critical mass of innovation social capital that will enable health services to address challenges they face.

## Supplementary Information

**Additional file 1.**

**Additional file 2.**

**Additional file 3.**

**Additional file 4.**

**Additional file 5.**

**Additional file 6.**

**Additional file 7.**

**Additional file 8.**

**Additional file 9.**

**Additional file 10.**

**Additional file 11.**

## Data Availability

All de-identified data is available in the QUT data repository (QUT - Research Data Finder).

## Abbreviations

EBP: Evidence-based practice
CFIR: Consolidated Framework for Implementation Research
IEBP: Implementing evidence-based practice
