# Supplementary material for: Impact of a health services innovation university program in a major public hospital and health service: a mixed methods evaluation
Source: Implement Sci Commun. 2022 Apr 25;3:46. doi: 10.1186/s43058-022-00293-3 (PMC9036712; doi:10.1186/s43058-022-00293-3)
Supplement: Supplementary file 1 — Additional file 1. [file 43058_2022_293_MOESM1_ESM.docx]

**Description of the program**

There are three core components of the program: delivery of post-graduate level units (subjects), student support services and innovation implementation support. The core components are summarised in Box 1.

Four university units (subjects) are completed part-time over two years in the fields of implementation science, health systems and cost-effectiveness analysis, with an applied workplace-based project in the final semester that consolidates students’ learning. Students were strategically selected from across the health service from clinical and non-clinical roles due to their demonstrated innovation and leadership skills. Student cohorts are comprised exclusively of staff from the health service and tuition fees are subsidized by the health service.

Box 1: Components of the Health Services Innovation program

**Units**

1. Implementation Science: Theory and Application in Health
2. Health Systems
3. Cost-Effectiveness Analysis for Healthcare Decision Making
4. Independent project

**Student Support Services**

- Enrolment completed for students
- Navigating a modern library
- Referencing skills
- Interpreting marking criteria
- Flexibility with assessment extension requests
- Twice-yearly individual student meetings at students’ workplaces

**Innovation implementation support**

- Bespoke matching of supervisors and students for project supervision
- Monthly drop-in clinics at students’ workplaces
- Knowledge into Practice workshops for students and alumni
- Personalised academic support specific to needs of workplace-based projects

The academic units were designed to deliver content in the prescribed order so that students could slowly build their confidence to plan, implement and evaluate new ideas or improvements to the health service. Implementation science is a field that uses theory and practice to provide guidance for successful health service program implementation, scale-up and longevity. In the Health Systems unit, students apply their theoretical knowledge of the outer implementation context to learn features that influence health service innovations or improvements, and understand what aspects are beyond their sphere of influence. In the cost-effectiveness analysis unit, students gain practical skills in evaluating the costs and health outcomes of an innovation or improvement in health services. In the independent project unit students consolidate their learnings, implement, or evaluate a workplace-based project and prepare a report or manuscript for publication in which they communicate findings. One unit is completed per semester, with face-to-face lectures delivered on the university campus in block-format over consecutive days. The aim of creating exclusive cohorts of students and bringing them together face to face is to facilitate internal relationship-building and peer support networks within the health service. Students usually complete the academic curriculum in two years.

Student support services and innovation implementation support is led by a university lecturer and researcher who simplifies the university experience for students. The academic is the single point of contact for students and solves problems immediately or triages their questions and requirements to the various contacts within the university. Consequently, the academic develops close relationships with the students and is capable of matching students with university supervisors for completion of the work-place based project. The health service also appointed a dedicated professional to the program: a Learning Coordinator. The purpose of this role is to facilitate the strategic support and alignment of students’ study and workplace-based projects within the health service. Innovation implementation support delivered through both these roles aims to transfer students’ learnings into real-world practice and build a network of innovators within the sponsoring health service. The additional program includes returning-to-study support, university seminars, specialized project advice, careful selection of university and health service supervisors for students’ projects, internal health service advocacy, coaching, establishing networks and an alumni. The additional two elements of support provided to students – both administrative and project-focussed across the two organisations – were included in the program as critical elements that ensured the application of the academic curriculum and tangible benefits to the health service.

More information can be found here: <https://www.aushsi.org.au/training/>
