# Supplementary material for: Impact of a health services innovation university program in a major public hospital and health service: a mixed methods evaluation
Source: Implement Sci Commun. 2022 Apr 25;3:46. doi: 10.1186/s43058-022-00293-3 (PMC9036712; doi:10.1186/s43058-022-00293-3)
Supplement: Supplementary file 2 — Additional file 2. [file 43058_2022_293_MOESM2_ESM.docx]

## **Scope and setting of study**

## *Scope*

The program is part of a broader Research Strategy (2017-2022) (1) within the health service that aims to increase health services research and value-based healthcare approaches. This study focuses on the impact of the program alone, not other concurrent health service initiatives.

## *Setting*

The health service is one of the largest in Australia and was established in 2011 (2). It has a local population catchment of 900 000 people, also delivers state-wide services and has an annual budget of $3 billion with a distinct and strongly-supported research strategy (1). There are five hospitals with inpatient and ambulatory capabilities; two of the facilities are tertiary/quaternary hospitals. A broad range of community and public health services are also provided (3).

1. Metro North Hospital and Health Service. Research Strategy 2017-2022. Brisbane: Metro North Hospital and Health Service; 2017.

2. Queensland Government. Metro North HHS Service Agreements Brisbane: Queensland Government; 2020 [Available from: <https://www.publications.qld.gov.au/dataset/metro-north-hhs-service-agreements>.

3. Metro North Hospital and Health Service. Health Service Strategy. Brisbane: Metro North Hospital and Health Service; 2017.
