# Supplementary material for: Impact of a health services innovation university program in a major public hospital and health service: a mixed methods evaluation
Source: Implement Sci Commun. 2022 Apr 25;3:46. doi: 10.1186/s43058-022-00293-3 (PMC9036712; doi:10.1186/s43058-022-00293-3)
Supplement: Supplementary file 3 — Additional file 3. [file 43058_2022_293_MOESM3_ESM.docx]

**Implementing Evidence-Based Practice (IEBP) Survey Methods**

A validated Implementing EBP (IEBP) quantitative survey informed by the work of Park et al. (1) was administered to the first and second cohorts of students, their managers and a control group of managers. As the program is designed to be implemented long-term, a quantitative approach was deemed feasible for evaluating annual changes at the health service’s operational level. Participants also had the option to respond to open-ended questions. The aim was to examine any potential differences between groups in use of evidence, implementation and evaluation. The IEBP survey instrument is available in Additional File 4.

All enrolled students from cohorts 1 and 2 (n = 60), and their direct line managers (n = 60) were selected to participate in the electronic IEBP survey. A control group of 60 managers were also invited to participate. the purpose of the control group was to understand what impact various levels of exposure to the program had on perceptions of EBP, as well as to gain an understanding across the health service of baseline EBP use of staff with no exposure to the program. These control managers were selected by identifying the level and discipline of each of the students’ line managers and then identifying randomly matched staff who were the same level and discipline from the full list of hospital and health service staff.

The electronic survey was built and administered via the software Key Survey. An email inviting participants to complete the survey was sent directly from a health service executive who was also an investigator in the research team. Reminders were sent within 4 weeks of the original invitation. The link to the electronic survey was embedded in the email.

The primary outcome in the quantitative analysis was self-reported implementation of EBP. Secondary outcomes were EBP self-efficacy, knowledge translation self-efficacy, comfort with EBP, intention to use EBP, and variables related to organizational context. Participant and outcome variables were analyzed descriptively. We intended to control for the independent variables using regression analyses however this was not possible due to low response rates. We believe it is also important to report our failure in recruiting an appropriate sample and that robust results are not available for this aspect of the methods.

1. Park JS, Moore JE, Sayal R, Holmes BJ, Scarrow G, Graham ID, et al. Evaluation of the "Foundations in Knowledge Translation" training initiative: preparing end users to practice KT. Implementation Science. 2018;13(63).
