# Supplementary material for: Impact of a health services innovation university program in a major public hospital and health service: a mixed methods evaluation
Source: Implement Sci Commun. 2022 Apr 25;3:46. doi: 10.1186/s43058-022-00293-3 (PMC9036712; doi:10.1186/s43058-022-00293-3)
Supplement: Supplementary file 4 — Additional file 4. [file 43058_2022_293_MOESM4_ESM.docx]

| 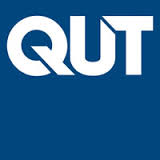 | **Survey** |
| --- | --- |
| **Evaluating the Effect of a Graduate Certificate (Health Services Innovation) program on Changes to Evidence Based Practice Culture and Implementation.**  **MNHHS Ethics Approval Number 48026**  **QUT Ethics Approval Number 1900000058** | |

1. What is your age? ______________
2. What is your gender

Female

Male

Other, please specify _______________

1. Within Metro North Hospital and Health Service, in which facility do you work primarily?

The Royal Brisbane and Women’s Hospital

The Prince Charles Hospital

Redcliffe Hospital

Caboolture Hospital/Kilcoy Hospital

Community and Oral Health

Mental Health Services

Other, please specify ____________________________________________________

1. What is/are your main area(s) of practice (select all that apply)?

Clinical

Policy and Planning

Project Management

Research

Team Leader

Director/Management

Clinical Education

Other, please specify

1. What is your current classification level and grade? E.g. A08, MO10, HP4, NG5?
2. How many years experience do you have in the health system?

Continuous – less than 1 year to 50 years or more

1. What is your highest qualification?

Secondary School

Bachelor Degree

Postgraduate Certificate/ Diploma

Masters

Doctorate

Other, please specify

1. Are/were you a current or past participant in the Graduate Certificate in Health Science (Health Service Innovation) program? *(Branching question – identifies “student” group)*

Yes

No

1. *(For students only)* What year did you commence the course? ____________________________
2. Are you the manager of a current/past participant in the course? *(Branching question – identifies “manager of students” group)*

Yes

No

**Evidence Definition**

*Throughout this survey when we use the term ‘evidence’ we are referring to the best available research evidence.*

**Self-efficacy to perform evidence-based management (EBM) activities**

1. Please rate your ability to perform each activity from 1 (terrible, I cannot do this at all) to 4 (not sure or I may be able to do this) to 7 (excellent, I can do this well).

|  | 1- Terrible | 2- Very Poor | 3- Poor | 4- Not sure | 5- Good | 6- Very good | 7- Excellent |
| --- | --- | --- | --- | --- | --- | --- | --- |
| a) Formulate a question to guide a literature search. |  |  |  |  |  |  |  |
| b) Effectively search the relevant literature for reliability and relevance. |  |  |  |  |  |  |  |
| c) Critically appraise the literature for reliability and relevance. |  |  |  |  |  |  |  |
| d) Critically appraise the strengths and weaknesses of different study designs. |  |  |  |  |  |  |  |

**Evidence Translation Self-efficacy**

1. Please rate your ability to perform each activity from 1 (terrible, I cannot do this at all) to 4 (Satisfactory; I am able to do this) to 7 (excellent, I can do this with excellence). A knowledge user is defined as an individual who is likely to use research evidence to make informed decisions about health policies, programs and/or practices.

|  | 1- Terrible | 2- Very Poor | 3- Poor | 4- Satisfactory | 5- Good | 6- Very good | 7- Excellent |
| --- | --- | --- | --- | --- | --- | --- | --- |
| a) I can conduct a needs assessment for knowledge users. |  |  |  |  |  |  |  |
| b) I can adapt research evidence to the needs of local knowledge users/stakeholders (e.g., clinicians, health care managers) |  |  |  |  |  |  |  |
| c) I can identify barriers and enablers to evidence uptake across different knowledge users. |  |  |  |  |  |  |  |
| d) I can identify strategies for evidence translation based on an assessment of barriers and enablers. |  |  |  |  |  |  |  |
| e) I can develop a strategy for monitoring use of evidence. |  |  |  |  |  |  |  |
| f) I can develop a strategy for evaluating relevant outcomes from use of evidence. |  |  |  |  |  |  |  |
| g) I can develop a strategy for sustaining use of evidence over time. |  |  |  |  |  |  |  |

**Research Utilisation and Comfort with Evidence**

1. Please rate your ability to perform each activity from 1 (Strongly disagree, I cannot do this at all) to 4 (neither agree or disagree) to 7 (Strongly agree, I can do this well).

|  | 1- Strongly disagree | 2- Disagree | 3- Somewhat disagree | 4- Neither agree nor disagree | 5- Somewhat agree | 6- Agree | 7- Strongly agree |
| --- | --- | --- | --- | --- | --- | --- | --- |
| a) I use evidence to inform decision-making in my current role |  |  |  |  |  |  |  |
| b) I am comfortable using evidence to inform my practice |  |  |  |  |  |  |  |

**Intention to use evidence**

1. Intention to use evidence. Please rate your ability to perform each activity from 1 (Strongly disagree, I cannot do this at all) to 4 (neither agree or disagree) to 7 (Strongly agree, I can do this well).

|  | 1- Strongly disagree | 2- Disagree | 3- Somewhat disagree | 4- Neither agree nor disagree | 5- Somewhat agree | 6- Agree | 7- Strongly agree |
| --- | --- | --- | --- | --- | --- | --- | --- |
| a) I expect to use high-quality evidence to help work through what I will discuss with managers, patients or clinicians during encounters with them. |  |  |  |  |  |  |  |
| b) I want to use high-quality evidence to help work through what I will discuss with managers, patients or clinicians during encounters with them. |  |  |  |  |  |  |  |
| c) I intend to use high-quality evidence to help work through what I will discuss with managers, patients or clinicians during encounters with them. |  |  |  |  |  |  |  |

**Organisational Context**

For each of the following statements, please rate the strength of your agreement with the statement, from 1 (strongly disagree) to 5 (strongly agree).

1. **Culture: Senior leadership/clinical management in your organization**:

|  | 1 - Strongly Disagree | 2 - Disagree | 3 - Neither Agree nor Disagree | 4 - Agree | 5 - Strongly Agree | Don't Know/Not Applicable |
| --- | --- | --- | --- | --- | --- | --- |
| a) reward clinical innovation and creativity to improve patient care |  |  |  |  |  |  |
| b) solicit opinions of clinical staff regarding decisions about patient care |  |  |  |  |  |  |
| c) seek ways to improve patient education and increase patient participation in treatment |  |  |  |  |  |  |

1. **Culture: Staff members in your organization**:

|  | 1 -Strongly Disagree | 2 - Disagree | 3 - Neither Agree nor Disagree | 4 - Agree | 5 - Strongly Agree | Don't Know/Not Applicable |
| --- | --- | --- | --- | --- | --- | --- |
| a) have a sense of personal responsibility for improving patient care and outcomes |  |  |  |  |  |  |
| b) cooperate to maintain and improve effectiveness of patient care |  |  |  |  |  |  |
| c) are willing to innovate and/or experiment to improve clinical procedures |  |  |  |  |  |  |
| d) are receptive to change in clinical processes |  |  |  |  |  |  |

1. **Leadership: Senior leadership/clinical management in your organization**:

|  | 1 - Strongly Disagree | 2 - Disagree | 3 - Neither Agree nor Disagree | 4 - Agree | 5 - Strongly Agree | Don't Know/Not Applicable |
| --- | --- | --- | --- | --- | --- | --- |
| a) provide effective management for continuous improvement of patient care |  |  |  |  |  |  |
| b) clearly define areas of responsibility and authority for clinical managers and staff |  |  |  |  |  |  |
| c) promote team building to solve clinical care problems |  |  |  |  |  |  |
| d) promote communication among clinical services and units |  |  |  |  |  |  |

1. **Measurement: Senior Leadership/clinical management in your organization**:

|  | 1 - Strongly Disagree | 2 - Disagree | 3 - Neither Agree nor Disagree | 4 - Agree | 5 - Strongly Agree | Don't Know/Not Applicable |
| --- | --- | --- | --- | --- | --- | --- |
| a) provide staff with information on performance measures and guidelines |  |  |  |  |  |  |
| b) establish clear goals for patient care processes and outcomes |  |  |  |  |  |  |
| c) provide staff members with feedback/data on effects of clinical decisions |  |  |  |  |  |  |
| d) hold staff members accountable for achieving results |  |  |  |  |  |  |

1. **Readiness for change: Opinion leaders in your organization**:

|  | 1 -Strongly Disagree | 2 - Disagree | 3 - Neither Agree nor Disagree | 4 - Agree | 5 - Strongly Agree | Don't Know/Not Applicable |
| --- | --- | --- | --- | --- | --- | --- |
| a) believe that the current practice patterns can be improved |  |  |  |  |  |  |
| b) encourage and support changes in practice patterns to improve patient care |  |  |  |  |  |  |
| c) are willing to try new clinical protocols |  |  |  |  |  |  |
| d) work cooperatively with senior leadership/clinical management to make appropriate changes |  |  |  |  |  |  |

1. **Resources: In general in my organization, when there is agreement that change needs to happen**:

|  | 1 - Strongly Disagree | 2 - Disagree | 3 - Neither Agree nor Disagree | 4 - Agree | 5 - Strongly Agree | Don't Know/Not Applicable |
| --- | --- | --- | --- | --- | --- | --- |
| a) we have the necessary support in terms of budget or financial resources |  |  |  |  |  |  |
| b) we have the necessary support in terms of training |  |  |  |  |  |  |
| c) we have the necessary support in terms of facilities |  |  |  |  |  |  |
| d) we have the necessary support in terms of staffing |  |  |  |  |  |  |

1. *(“Student” group only)* What learnings have you taken and applied from the course in your day to day practice? _____________________________________________________
2. In your opinion, does the facilitation of a Graduate Certificate in Health Science (Health Service Innovation) to enrolled MNHHS staff lead to better implementation of evidence-based practice within the HHS? ______________________________________________________
3. In your opinion, what are the enablers of and barriers to implementation of evidence-based practice within MNHHS? ____________________________________________
4. Do you have any feedback you would like to share about the Graduate Certificate in Health Science (Health Service Innovation) program? For example, highlights, things that you would change ______________________________________________________________
5. (“Managers of students” group only) The research team would like to interview managers of the first cohort of students who commenced in 2018. The purpose of these interviews is to further understand expectations of the Graduate Certificate (Health Services Innovation) course and your opinion on Metro North HHS’ capacity to implement evidence-based practice. Please indicate your willingness to participate in an interview *(Branching question – if “yes” then question 26 will be displayed)*

Yes

No

1. *(People who respond “yes” to Q25 only)* Please provide your email address so we can contact you to arrange an interview. Your email address will not be stored with your response from this survey ________________________

Thank you for your participation in this study. Your responses will help to guide the ongoing development of the Graduate Certificate program. If you would like feedback on the results of this study please contact Elizabeth Martin at elizabethkate.martin@qut.edu.au
