# Supplementary material for: Impact of a health services innovation university program in a major public hospital and health service: a mixed methods evaluation
Source: Implement Sci Commun. 2022 Apr 25;3:46. doi: 10.1186/s43058-022-00293-3 (PMC9036712; doi:10.1186/s43058-022-00293-3)
Supplement: Supplementary file 5 — Additional file 5. [file 43058_2022_293_MOESM5_ESM.docx]

| 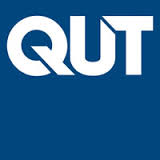 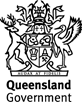 | **Interview Facilitator’s Guide** |
| --- | --- |
| **Evaluating the Effect of a Graduate Certificate (Health Services Innovation) program on Changes to Evidence Based Practice Culture and Implementation.**  **MNHHS Ethics Approval Number xxx**  **QUT Ethics Approval Number xxx** | |

**Participants (interview facilitated by)**

CI Janet Davies

**Informed Consent to Participate:**

Participants will be asked to sign a Participant Information and Consent Form prior to the commencement of the interview, including acknowledgement of the audio recording. Phone participants will be asked to email the signed copy to the interviewer prior to the interview. Participants will be given the opportunity to ask any questions they might have about the study prior to providing consent.

**Demographics Survey:**

Hand out the paper-based demographics survey to be completed prior to commencement of the interview.

**Introduction**

“I’m ______ and I’m interviewing representatives of Metro North Hospital and Health Service as part of the evaluation of the Graduate Certificate in Health Sciences Innovation program. We are looking for your perspective on the current capacity of MNHHS to implement evidence based approaches to evidence based practice, as well as your expectations of the Graduate Certificate course and whether the course is meeting those expectations.

This meeting/ phone call is being recorded so that an accurate record of your comments can be kept. The Graduate Certificate teaching staff will not know which comments can be attributable to which participant. No identifying information will be included in the evaluation report or any publications. Your participation is voluntary. It is expected that this interview will take up to one hour and you are welcome to skip any question that you do not wish to answer, or to leave at any time.”

**Questions:**

1. How would you describe MNHHS’ current organisational capacity to implement evidence-based approaches to evidence-based practice and evaluation? ‘Capacity’ refers to knowledge, skills, awareness, supportive infrastructure and time, and ‘evidence’ refers to academic research evidence such as that published in peer-reviewed journals, reports of rigorous evaluation including economic evaluations, and clinical guidelines. (Measurement of MNHHS Knowledge Translation Capacity, Strategy 3.1)

**Note to interviewer:* If further explanation is needed use this prompt

When we talk about organisational capacity for evidence-based practice we are referring to organisational structures and culture which influence the uptake of research knowledge and evidence-based practice such as: having the knowledge, skills, awareness, supportive infrastructure and time to review research evidence, to access training, to have appropriate resources, have access to leaders and experts, and to be able to implement practices which are based on research findings.

**If further examples are needed use this prompt:*

For example is there sufficient time to be able to review and read the latest research in your area, do you have access to the resources you need to be able to implement changes to your practice based on research findings, are leaders or experts readily accessible, and is training available and accessible.

1. Are you aware of groups/teams/units within MNHHS that are using evidence-based approaches to implement evidence into practice (Measurement of MNHHS Knowledge Translation Capacity, Strategy 3.1)
2. What were your expectations of the Graduate Certificate in Health Science Innovation course? What did you hope it would achieve in the short-term and long-term for MNHHS and QUT? (Course Evaluation, Strategy 1.1)
3. Has the course met those expectations to date? Please explain (Course Evaluation, Strategy 1.1)
4. What enablers and barriers have impacted on the capacity of MNHHS to implement evidence-based approaches to evidence-based practice? (Measurement of MNHHS Knowledge Translation Capacity, Strategy 2.1)
5. What do you think could be changed for the next round of this course to better meet the needs of participants/ MNHHS? (Course Evaluation, Strategy 1.1)

**Conclusion:**

Thank you for your participation. Your comments will help to shape the structure and content of the Graduate Certificate in Health Sciences Innovation course for future cohorts. If you would like to add any additional comments or clarify any statements you will have the chance to do so within the next week by contacting me by phone or email *(provide the participant with a business card/ contact information).* If you choose to withdraw your consent to participate within one week of this interview, on request, any identifiable information obtained from you will be destroyed.
