# Supplementary material for: Impact of a health services innovation university program in a major public hospital and health service: a mixed methods evaluation
Source: Implement Sci Commun. 2022 Apr 25;3:46. doi: 10.1186/s43058-022-00293-3 (PMC9036712; doi:10.1186/s43058-022-00293-3)
Supplement: Supplementary file 6 — Additional file 6. [file 43058_2022_293_MOESM6_ESM.docx]

| 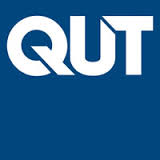 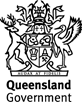 | **Semi-Structured Focus Group Guide** |
| --- | --- |
| **Evaluating the Effect of a Graduate Certificate (Health Services Innovation) program on Changes to Evidence Based Practice Culture and Implementation.**  **MNHHS Ethics Approval Number 48026**  **QUT Ethics Approval Number 1900000058** | |

**Participants:**

Cohort One students of the Graduate Certificate program

**Investigator and Facilitator:**

Dr Olivia Fisher

**Informed Consent to Participate:**

Focus group participants will be asked to sign a Participant Information and Consent Form prior to the commencement of the interview, including acknowledgement of the audio recording. Participants will be given the opportunity to ask any questions they might have about the study prior to providing consent.

**Demographics Survey:**

Hand out the paper-based demographics survey to be completed prior to commencement of the interview.

**Introduction**

“I’m ______ and I’m conducting focus group interviews with representatives of Metro North Hospital and Health Service as part of the evaluation of the Graduate Certificate in Health Sciences Innovation program. We want to understand your perspective on what the evidence-based practice culture was like before the Graduate Certificate course, your expectations of the course, whether the course met those expectations, enablers and barriers to your participation, highlights of the course, and any other feedback you would like to provide.

This focus group is being recorded so that an accurate record of your comments can be kept. No identifying information will be included in the evaluation report or any publications. Your participation is voluntary. It is expected that this interview will take up to one hour and you are welcome to skip any question that you do not wish to answer, or to leave at any time.”

**Questions:**

1. Describe your individual capacity prior to the course to implement evidence-based practice? ‘Capacity’ refers to knowledge, skills, awareness, supportive infrastructure and time, and ‘evidence’ refers to academic research evidence such as that published in peer-reviewed journals, reports of rigorous evaluation including economic evaluations, and clinical guidelines. (Measurement of MNHHS Knowledge Translation Capacity, Strategy 3.1)

**Note to interviewer: If further explanation is needed use this prompt*

When we talk about individual capacity for evidence-based practice we are referring to: having the knowledge, skills, awareness, supportive infrastructure and time to review research evidence, to access training, to have appropriate resources, have access to leaders and experts, and to be able to implement practices which are based on research findings.

*If further examples are needed use this prompt:*

For example is there sufficient time to be able to review and read the latest research in your area, do you have access to the resources you need to be able to implement changes to your practice based on research findings, are leaders or experts readily accessible, and is training available and accessible.

1. How much do you feel that you were already implementing evidence-based approaches to evidence based practice, good evaluation practices, and/or the principles of implementation science prior to your participation in the course? If appropriate, please give an example (Measurement of MNHHS Knowledge Translation Capacity, Strategy 3.1)
2. What were your expectations of the graduate certificate course when you enrolled? (Course Evaluation, Strategy 1.1)
3. Has the course met those expectations to date? Please explain (Course Evaluation, Strategy 1.1)
4. What would say are the key learning(s) that participating in the course has provided you? (Course Evaluation, Strategy 1.1)
5. Have you applied your learnings from the course? If so give some examples, if not how might you apply your knowledge in future? (Course Evaluation, Strategy 1.1)
6. What enablers and barriers have impacted on your participation in the course? (Course Evaluation, Strategy 1.1)
7. What do you think could be changed for the next round of this course to better meet the needs of participants/ MNHHS? (Course Evaluation, Strategy 1.1)

**Conclusion:**

“Thank you for your participation. Your comments will help to shape the structure and content of the Graduate Certificate in Health Sciences Innovation course for future cohorts. If you would like to add any additional comments or clarify any statements you will have the chance to do so within the next week by contacting me by phone or email *(provide the participant with a business card/ contact information).* If you choose to withdraw your consent to participate within one week of this interview, on request, any identifiable information obtained from you will be destroyed.”
