# Supplementary material for: Impact of a health services innovation university program in a major public hospital and health service: a mixed methods evaluation
Source: Implement Sci Commun. 2022 Apr 25;3:46. doi: 10.1186/s43058-022-00293-3 (PMC9036712; doi:10.1186/s43058-022-00293-3)
Supplement: Supplementary file 7 — Additional file 7. [file 43058_2022_293_MOESM7_ESM.docx]

**Data Collection Procedures**

Recruitment and data collection occurred between June and October 2019, 18 months after program delivery had commenced.

Executive Interviews

Structured interviews with members of the health service and university executive leadership were conducted to identify their perceptions of changed capacity across the organization. Interview questions are in Additional File 5. Participants were selected and invited to an interview based on their previous strategic involvement with the program either in a conception, funder or approval role. Executives’ assistants were emailed an invitation directly from a health service executive who was also an investigator in the research. An appointment time for the interview was made and the data collection procedure henceforth followed the interview guide in Additional File 5.

Student Focus Groups

Semi-structured focus groups were conducted with students from the first cohort to identify preliminary changes in health service capacity during their 18 months of participation. The focus group guide is available in Additional File 6. The first cohort of students was selected because of their deep knowledge of the program and willingness to provide feedback and engage in deep thought regarding program improvements and sustainability. Students were invited to participate via email from the chief investigator of the research evaluation. Proposed times were suggested for focus groups located at each campus of the health service and additional focus groups were arranged as necessary. Data collection procedures commenced for each focus group as outlined in the focus group guide in Additional File 6.

Interviews with Students’ Managers

Interviews were planned to be conducted with approximately ten managers of students enrolled in the program. The intent was to evaluate the implementation and short-term impact of the program from the perspective of health service staff affected by, but not directly involved in the program and associated decision-making. Managers were asked to indicate on the IEBP survey (see Additional files 3 and 4) whether they would be willing to participate in an interview. However, no managers agreed to participate in the interviews and in the interests of transparency, our failure to execute this method is important to report.
