# Supplementary material for: Impact of a health services innovation university program in a major public hospital and health service: a mixed methods evaluation
Source: Implement Sci Commun. 2022 Apr 25;3:46. doi: 10.1186/s43058-022-00293-3 (PMC9036712; doi:10.1186/s43058-022-00293-3)
Supplement: Supplementary file 11 — Additional file 11. [file 43058_2022_293_MOESM11_ESM.docx]

**Detailed results**

Executive interview participants have been reported in the manuscript. In the student focus groups, six participants were from the allied health discipline, three from nursing, one from medicine and one was from a multi-discipline professional stream. In terms of work responsibilities, one participant worked in a hybrid management-clinician role. The other ten participants were former clinicians working as either as discipline leaders or project managers. IEBP survey responses were received from 13 students, eight managers of students, and seven control managers. One participant was both a student and the manager of a student. This participant’s responses have been included with the student data due to their exposure to the program (Table 1).

*Themes*

Four overarching themes emerged from the qualitative data.

Realization of knowledge gaps

University and health service executives had similar views on the health service’s pre-program capacity. There had been a constant flow of new projects within the health service, however there was a lack of rigor around implementation process and evaluation, meaning:

*scalability and sustainability is poor* (executive interview 1).

*… the other thing is because we don’t have huge strengths around evaluation methodology, we’re not very good at proving back to the system, that implementing that change is driving a measurable benefit ...* (executive interview 1)

Table 1: Characteristics of Implementing Evidence Based Practice (IEBP) survey participants

|  | **Students n = 13** | **Managers of student n = 8** | **Control Manager n = 7** |
| --- | --- | --- | --- |
| Gender | Female n = 8  Male n = 7 | Female n = 3  Male n = 4 | Female n = 5  Male n = 2 |
| Year of course commencement | Year 2018 n = 8  Year 2019 n = 5 | N/A | N/A |
| Position classification | Senior administrator n = 1  Senior allied health n = 6  Director allied health n = 2  Medical director n = 1  Medical officer n = 2  Dental specialist n = 1 | Senior administrator n = 1  Senior director allied health n = 2  Director nursing n = 3  Senior medical officer n = 1  Missing n = 1 | Senior administrator n = 1  Executive n = 1  Director allied health n = 1  Senior director allied health n = 1  Dental director n = 1  Medical officer n = 2 |
| Areas of practice  (participants could select more than one) | Clinical = 7  Research = 1  Team leader = 1  Director/ Management = 2  Workforce development officer = 2 | Clinical = 2  Director/ Management = 6 | Clinical = 2  Director/ Management = 4  Engineering and building maintenance = 1 |
| Average years’ experience in health system | 18 | 27.4 | 22 |

Health service executives were aware of a small number of expert individuals within the organisation who were already using or doing EBP and using implementation science to effect change independent of the program:

*we … struggle once that champion goes onto the next the hobby* (executive interview 1)

In the student focus groups and IEBP survey open-ended responses, students reported that many projects implemented pre-program lacked a framework that supported sustainability, engaged stakeholders, and led to frustration for individuals and across the health service:

*So, prior to this I thought I was ticking the box. I now know I wasn’t ticking the box ...* (student focus group 2)

*Before the course started we did … a change to all of [service] provision and … you could say it was not done well … and I think that is very much due to the fact that we didn't engage with the stakeholders sufficiently particularly like consumer groups … we did do some focus groups with them some of the feedback that they came back with was easy to brush off if we felt it was irrelevant or not based on a lot of truth…. I definitely think if we did that again, I would do it much differently after having done the course.* (student focus group 1)

*My team… made lots of assumptions on what people thought, we didn’t actually ask them, or we didn’t spend as much time. I mean we said ‘these are the key stakeholders’ and we might go and meet with those key stakeholders, but we went in there with an agenda, that was our agenda, not their agenda ...* (student focus group 4).

Students reported that although prior to the program they knew what EBP was, there were gaps in their understanding of how to translate and disseminate evidence:

*... we did have I guess an approach as managers within that service … of well too bad this is what you’ve got to do, that was kind of the approach taken … so we didn’t have no success but we almost had resentment building … and you’re going to have people who remember the last time you tried to make a change and were still, in a word, pissed.* (student focus group 5)

Increased individual and network capacity

Students and executives both thought that the individual capacity of students, as well as the network capacity, had increased as a direct result of their participation in the program. Executives observed an increase in knowledge and culture change within the student group in terms of enthusiasm and improved implementation planning:

*… how much potential this cohort has for driving beneficial change was hearing them pitch the projects that they were already undertaking or planning to undertake in various stages and, and seeing the potential benefit from that …* (executive interview 5)

In the IEBP survey, students and managers of students who had been exposed to the course felt certain that participation resulted in increased EBP knowledge, shared language and skills (Table 2):

*Yes, the grad cert provides the skills and framework to implement sustainable practice changes within a complex and often multifaceted environment.* (student IEBP survey comment)

Managers of students reported that they had observed the students using enhanced skills and techniques in their day-to-day work:

*I have experience of line managing three participants of the Grad Cert in Health Service Innovation and have witnessed those staff grow, use new techniques and explore ways to improve, enhance and change service delivery and models of care.* (manager IEBP survey comment)

However, across the three IEPB survey groups there were no significant differences in any of the EBP variables measured (Table 2). Students, students’ managers and control managers all rated highly their intention to use evidence, their comfort with using evidence to inform practice, and their use of evidence (Table 2). Despite this, students reported that the program increased their individual capacity, particularly in terms of confidence and their credibility within the organization. They could now build the sustainability of improvement projects and interventions through structures, frameworks and processes. They were also more aware of the reality and challenges of sustaining a change in a health service:

*… I have consciously stopped, and thought hang on, am I, from an implementation point of view, covering everything that I need to do at different phases of implementation and like I said I have consciously thought about different frameworks and applying those. A lot of the stuff has so far just been in the planning for implementation rather than the actual … In saying that, we had successfully implemented a new model of care and service in [facility name] and that project finished at the end of June last year. But we’re now transitioning it to business as usual, which is actually throwing up some unexpected challenges and as I’ve been dealing with those and thinking how do we avoid this happening again, I’ve been reflecting back again on the implementation science frameworks and things …* (student focus group 3)

Table 2: Outcome variable scores, Implementing Evidence Based Practice (IEBP) survey

|  | **Possible range** | **Cohort 1 n** | **Cohort 1 mean (SD)** | **Cohort 2 n** | **Cohort 2 mean (SD)** | **Students’ managers n** | **Students managers mean (SD)** | **Control managers n** | **Control managers mean (SD)** |
| --- | --- | --- | --- | --- | --- | --- | --- | --- | --- |
| Self-efficacy to perform evidence-based management activities | 4 - 28 | 8 | 21.5 (2.8) | 5 | 18.8 (2.2) | N/A | N/A | N/A | N/A |
| Evidence translation self-efficacy | 7 - 49 | 8 | 37.8 (4.0) | 5 | 34.4 (2.6) | 8 | 35.3 (4.1) | 7 | 33.4 (11.7) |
| Research utilization and comfort with evidence in current role | 1 - 7 | 8 | 6.1 (0.8) | 5 | 5.8 (2.6) | 8 | 6.3 (0.5) | 7 | 6.6 (0.5) |
| Research utilization and comfort with evidence informing practice | 1 - 7 | 8 | 6.1 (0.6) | 5 | 6.0 (1) | 8 | 6.4 (0.5) | 7 | 6.7 (0.5) |
| Intention to use evidence | 3 - 21 | 8 | 18.5 (1.7) | 5 | 17.4 (3.6) | 8 | 18.4 (1.6) | 7 | 19.4 (1.9) |
| Positive culture amongst senior leadership/ clinical management | 3 - 21 | 8 | 11.8 (1.8) | 4 | 13.3 (1.7) | 8 | 12.6 (1.3) | 6 | 11.7 (3.4) |
| Positive culture amongst staff members | 4 - 20 | 8 | 15.1 (2.4) | 4 | 16.3 (2.1) | 8 | 15.6 (2.3) | 6 | 16.7 (2.8) |
| Senior leadership/ clinical management lead teams effectively | 4 - 20 | 8 | 14.1 (1.8) | 4 | 13.3 (1.7) | 8 | 14.6 (3.9) | 6 | 14.8 (2.9) |
| Senior leadership/ clinical management measure outcomes | 4 - 20 | 8 | 13.1 (2.2) | 3 | 15.3 (3.1) | 8 | 13.8 (3.6) | 7 | 14.9 (3.2) |
| Readiness for change lead by cooperative opinion leaders | 4 - 20 | 7 | 15.7 (2.1) | 5 | 16.6 (3.2) | 8 | 16.0 (1.7) | 7 | 15.4 (2.4) |
| Change is supported with resources | 4 - 20 | 8 | 10.4 (2.9) | 5 | 11.8 (3.2) | 8 | 10.5 (3.3) | 7 | 10.3 (3.2) |

Cohort 1 commenced February 2018

Cohort 2 commence February 2019

n: number of respondents

SD: Standard Deviation

N/A: Not applicable because managers not asked question

Promising, but early days

Executives reported that they their long-term vision to develop a critical mass of skilled individuals who would be able to enact “proactive, strategic, planned” change rather than “reaction to crisis” was not achieved yet. All participants consistently expressed that the preliminary impact of the program was promising but acknowledged that it was a long way from achieving the desired large scale cultarrure change:

*… and so the medium and the long term strategic view on this is to say we’ve got to create that critical mass inside the organization that it becomes the core way we do business as opposed to having individual talent that are actually being able to demonstrate and apply this inside the organization.* (executive interview 4)

*I don't think that's going to mature for a number of years yet because … this is a huge health service and these are a small number of staff you know a total of 60 staff out of close to 20 000 staff, you're not going to change things immediately.* (executive interview 3)

Students believed that the program would eventually change the organization’s culture and build organization capacity, but they acknowledged that “it’s early days”. With their new knowledge and realization of how implementation could be improved, students were frustrated at not seeing the organization’s culture change faster:

*I think it’s frustrating now watching the time that’s invested at an organizational level sometimes into projects that we can now see holes in from the start …* (student focus group 2)

In the open-ended IEBP survey responses, students, managers and control managers varied greatly in their opinions as to whether the program leads to better implementation of EBP in the organisation. Most control managers in the IEBP survey (85.7%; n=6) stated that they were unsure whether the program would have an impact on EBP within the health service, in contrast to those directly exposed to the program feeling certain about the long-term change:

*We have lots of enthusiastic staff with great ideas and hopefully increasingly, the skills to bring about change.* (manager IEPB survey comment)

The development of a shared language and approach to health services innovation were viewed as promising signs of organizational change:

*I find what’s the most beneficial sometimes as a clinician moving into … into business talk if you like, is the understanding of the terminology and the language which then really helps when you’re writing those proposals… from a financial, you know … how to frame it, so that you meet the finance and business interests as well as demonstrate the clinical benefits of it as well.* (student IEBP survey comment)

Tangible changes that students observed in the organization were around other staff being receptive of their advice, and increased success in embedding language and processes throughout the organization:

*… providing some additional advice and support with our department research coordinators. Just I guess trying to embed some of those concepts and making sure that they’re thinking that way and that the practicalities of implementation science are considered.* (student focus group 2)

*… we’re trying to embed a little bit of a translating research into practice framework bent into the some of the submissions that are going.* (student focus group 2)

A student found that this new way of working and the subsequent increase in rigor, was noticed by their executive director when the student presented the findings from their project:

*And it was interesting when we presented that to exec, they suddenly went this is amazing … we were given the time to do it properly and that set the standard for future projects because you know that’s what we do. There’s now an understanding the value of the rigor and an endorsement that that’s the right way to do it and my executive director actually refers to the fact that I went to Uni and the value of that, which is great.* (student focus group 4)

Organizational support in theory, barriers in practice

Within the health service, participants across all groups, regardless of their exposure to the program felt that executive leadership support for EBP was vital, and a key enabler of the use of EBP within the service. However, substantial barriers to the use of robust EBP processes were reported in practice:

*I think that the executive have made it clear that that they want the health service to be based on evidence, they want decisions to be informed by evidence, they want changes to be evaluated with quality of evidence. And I think this is a huge enabler for EBP and for doing effective safe efficient clinical work.* (executive interview 5)

*An enabler would be the support of the [service] executive and the provision of strong direction to those who are blockers.* (control manager IEBP survey comment)

The networks and communication that evolved because of the program, both within the health service and between the health service and the university, were considered a strong enabler of increased capacity to implement EBP. The program enabled greater interconnectedness:

*One of the greatest strengths our guys rave about is the networks they’ve built across the [multiple health service campuses] by coming together as a cohort.* (executive interview 1)

*… one of the ancillary benefits has been that it's bought two orgs together. We've got a better understanding of each other as part of this. We've developed stronger, more trusting relationships ...* (executive interview 7

The workforce in the health service was identified as both a challenge and facilitator of the program’s success. Participants described most of the workforce as being comfortable with current practice and resistant to innovation. There was great difficulty in changing culture:

*You know we’re like rubber bands, you know we're much more comfortable going back to our comfortable shape. So, if you let pressure off we'll go back to doing what we've always done the way we've always done it because we're creatures of habit and we're comfortable doing the stuff that we were trained, that we'd always done and we've always done what everyone else in the herd is doing.* *So, I think our innate sort of conservative nature by and large as a workforce means we don't adopt change easily.* (executive interview 3)

*Some areas are risk averse and see all change as a threat rather than opportunity.* (student IEBP survey comment)

Time and budgetary constraints, and lack of knowledge, were raised by students, and managers as key barriers to the use of robust implementation processes within the health service. While some students were in roles where they had access to project time and funds, this was seen as the exception, rather than the norm. In the IEBP survey, the mean scores for the access to resources measure ranged between 10.29 and 11.80 between groups out of a possible 20 points (Table 2).

Speaker 1 [Facilitator]: *… do you have sufficient resources, do you have sufficient time, has any of that changed …*

Speaker 3: *No*

Speaker 4: *Has the organization given us more capacity …*

Speaker 3: *If I sleep less*. (student focus group 2)

*A focus on finance means that EBP cannot be implemented unless cost neutral or profitable, or unless existing staff expend personal time and energy to provide the service.* (control manager IEBP survey comment)

In the interviews, university executives focused on “building a learning organization that is able to respond”. This responsiveness of the university in delivering a bespoke program was identified as an important enabler for the program’s success:

*… one of the things we’ve done well is be adaptive; so as long as we remain adaptive then we will change what needs to be changed as we go*. (executive interview 1)

*… I'm really glad that we were able to respond to that quickly and produce something that was more fit for purpose for this group … I've been delighted by the responsiveness of us, but I think that that's a principle that needs to continue … So, there's a bit of a balance between how you keep the broad structure, and how it's refreshed and how it meets the needs of individual groups.* (executive interview 7)
